# Heart Failure Trends in Paraíba: Earlier Diagnosis or Better Treatment? - That is One of the Questions

**DOI:** 10.36660/abc.20190898

**Published:** 2020-02

**Authors:** Ana Teresa Timóteo

**Affiliations:** 1 Santa Marta Hospital, Centro Hospitalar Lisboa Central, Lisboa - Portugal; 2 NOVA Medical School, Lisboa, Lisboa - Portugal

**Keywords:** Heart Failure/physiopathology, Heart Failure/mortality, Heart Failure/epidemiology, Comorbidity, Heart Failure/trends, Hospitalization, Health Care Costs

Heart Failure (HF) is a clinical syndrome with high prevalence, morbidity and mortality worldwide.^1-7^ It is also one of the main causes for hospital admissions with elevated direct and indirect costs.^1-7^ The prevalence of HF is expected to increase due to the increase in cardiovascular risk factors in the general population and to the increase in life expectancy. Elderly patients are more prone to the development of HF, as well as of admissions for HF. For that reason, an increase in the economic burden of HF is expected in the next decades.^1-7^

Previously published data showed, however, some epidemiological differences according to the world region, when comparing developing and developed countries. Recently published data from the European Society of cardiology Heart Failure Long-term Registry, a registry that included a wide spectrum of countries with very different socio-economic backgrounds, from Southern, Western, Northern and Eastern Europe, as well as Middle East and Northern Africa, showed significant between-region differences in baseline characteristics, clinical characteristics and also treatment and outcomes in patients with acute and chronic HF.^8^

Brazil is a very large country, with huge disparities between regions and thus, it was important to perform a regional study.

The article by Fernandes et al,^9^ published in this journal, is a retrospective study on epidemiological data obtained between 2008 and 2017, based on DATASUS database, a population database.^9^ They studied data specifically from the state of Paraíba, a region considered by the authors to be a developing state, compared to other parts of Brazil and they put the results into perspective by comparing with them with data from the entire country. Heart failure was the first cardiovascular cause of hospital admissions, both in Paraíba (29.4%) and Brazil (21%). There was a significant 62% decrease in hospital admissions and 37.5% in absolute numbers of hospital mortality due to heart failure in Paraíba, from 2008 to 2017. However, in-hospital mortality rates increased by 65.1%, from 6.6% to 10.9%. An increase in hospital length of stay of 44% was also observed. In absolute values, the authors found a non-significant reduction in death by heart failure, which was however significant when analyzing mortality rate, with a decline of 10.7%, being 14.0/100,000 inhabitants. The same trends were also observed in the general data from Brazil, but the magnitude of change was much higher in Paraíba.

This paper raises many important questions, which should be further addressed in subsequent studies. The reduction in hospital admissions might be the main explanation for the reduction in hospital mortality in absolute values. However, the increase in hospital mortality rate is an indirect sign that patients admitted were probably in worse clinical condition. The greatest improvement in this state, compared to Brazil, might be explained by a more sustained improvement in living conditions in Paraíba in the last decade, compared to others that are more developed and probably did not improve much in the last years, because the potential for improvements is higher in developing states. The authors did not show these specific data - was there a more significant increase in gross domestic product in Paraíba compared to other states?

The European study showed that in North Africa, the proportion of women is higher when compared to other groups, with younger patients, with less hypertension but more diabetes and smokers.^8^ The ischemic etiology was much less frequent, ejection fraction was more preserved and patients were less treated.^8^ In this registry, 1-year all-cause mortality was 23.6% for acute HF patients and 6.4% for chronic HF patients and 1-year hospitalization was 18.7% and 9.9% respectively. In North Africa, higher all-cause mortality was observed (15.6%) and lower hospitalization rates (10%) in the chronic HF group. Being from North Africa was an independent predictor of all-cause mortality in the acute HF group of up to 2.7 times compared to Southern Europe. The higher death rates observed were partially attributed to the much less frequent use of guideline-recommended medical therapies for HF with reduced ejection fraction, a problem shared by other low- and middle-income countries and regions, or by differences in the hospital admission criteria, also recently reported.^8,10,11^ In Brazil, substantial between-region differences are expected regarding demographic characteristics, clinical characteristics and treatment, when we compare developed and developing regions, which can explain some of the results obtained in mortality and hospitalization rates.

For that reason, the results presented for the state of Paraíba require additional information for better interpretation, which in some cases contradict worldwide data projections.^1,2^ Few studies have evaluated the different trends in HF with reduced ejection fraction (HFrEF) and preserved ejection fraction (HFpEF), but HFpEF might be dominant in the coming years, because in the past 20 years, trends have been reported on the increasing proportion of patients with HFpEF and relatively stable/decreasing rates of HFrEF^2^. In fact, the increase in HF prevalence might not be related to an increase in incidence. The aging of the population, together with improved HF survival, particularly in HFrEF, due to advancements in treatment is a likely explanation.^2^ In addition, prevention programs might be reducing the incidence, with lower severity and better treatment of ischemic heart disease.^2^ However, risk factors for coronary artery disease are still increasing. Thus, a reduction in HFrEF is expected, as well as an increase in HFpEF with consequent more cases of HF admissions due to HFpEF, with a lower mortality compared to HFrEF.^2^

The reduction in hospital admissions must also be clarified. Information regarding HF disease management program availability or the use of implantable devices (involving patient characteristics but also resource availability and reimbursement structure) can explain that reduction.^1,3^ Another possible explanation is that HF patients might be detected earlier in the course of disease, and with that, premature hospital admissions can be avoided. If they are treated according to guidelines, this can also delay and reduce hospital admissions and mortality.^1^

Early diagnosis and optimal treatment are important quality indicators for the treatment of HF, and this is also a question to be addressed. Is the medical assistance in Paraíba significantly different from that in the rest of the country? Is it mostly private practice or a public system of health care? What is patient accessibility to healthcare like and what were the improvements obtained (if any) in the last years? If feasible, all those questions about socio-economic conditions and healthcare data should be analyzed from 2008 to 2017 to see what the trend is and to better identify the main specificities that require investment.
